# Adiposity Criteria in Assessing Increased Cardiometabolic Risk in Prepubertal Children

**DOI:** 10.3389/fendo.2019.00410

**Published:** 2019-06-26

**Authors:** Tuomo Tapani Tompuri, Jarmo Jääskeläinen, Virpi Lindi, David Elliot Laaksonen, Aino-Maija Eloranta, Anna Viitasalo, Tomi Laitinen, Timo Antero Lakka

**Affiliations:** ^1^Department of Clinical Physiology and Nuclear Medicine, University of Eastern Finland and Kuopio University Hospital, Kuopio, Finland; ^2^Institute of Biomedicine/Physiology, School of Medicine, University of Eastern Finland, Kuopio, Finland; ^3^Sense4Health Ltd., Kontio, Finland; ^4^Department of Pediatrics, Kuopio University Hospital and University of Eastern Finland, Kuopio, Finland; ^5^Department of Internal Medicine, Kuopio University Hospital, Kuopio, Finland; ^6^Kuopio Research Institute of Exercise Medicine, Kuopio, Finland

**Keywords:** body fat percentage, body mass index, diagnostic accuracy, obesity, overweight, sensitivity, specificity, waist-to-height ratio

## Abstract

**Objective:** Adiposity induces the clustering of cardiometabolic risk factors, and pediatric adiposity is a better indicator for adulthood cardiometabolic diseases than pediatric metabolic syndrome. However, the observed prevalence of pediatric adiposity depends on the methods and cut-points used. Therefore, we aimed to define diagnostic criteria for adiposity which enable more valid identification of prepubertal children at increased cardiometabolic risk.

**Methods:** The participants were 470 prepubertal children (249 boys) aged 6–8 years. The measures of adiposity included body mass index—standard deviation score (BMI-SDS), waist-to-height ratio (WHtR) and body fat percentage (BF%) assessed by bioelectrical impedance analysis (BIA) and dual-energy X-ray absorptiometry (DXA). Criteria for adiposity were determined by increased cardiometabolic risk. Cardiometabolic risk factors which correlated with BF% assessed by DXA in the upper but not lower half of BF% (serum insulin and plasma high-density lipoprotein cholesterol, triglycerides, gamma-glutamyl transferase, high-sensitivity C-reactive protein and uric acid) were included in the cardiometabolic risk score (CMS). We computed receiver operating characteristics curves for the measures of adiposity using the ≥90th percentile of CMS as a measure of increased cardiometabolic risk, and local regression curves were graphed to demonstrate the associations of the measures of adiposity with CMS.

**Results:** In girls, WHtR of 0.445 (area under curve 0.778, its 95% confidence interval 0.65–0.91, sensitivity and specificity 0.73) and BF% of 19.5% assessed by BIA (0.801, 0.70–0.90, 0.73) were the best overall criteria for increased cardiometabolic risk. In boys, BMI-SDS of 0.48 (0.833, 0.75–0.92, 0.76) was the best overall criterion for increased cardiometabolic risk. While local regression curves in girls showed that WHtR of 0.445 corresponds well to a point where CMS began to increase, in boys local regression curves suggest that CMS began to increase even at a lower level of BMI-SDS than 0.48. Moreover, the diagnostic ability of the measures of adiposity to exclude increased cardiometabolic risk was poorer than the ability to detect it.

**Conclusions:** In general, the measures of adiposity have sufficient diagnostic accuracy to be utilized as the screening tool for increased cardiometabolic risk. The observed cut-points for adiposity were lower than the traditional cut-points for adiposity.

## Introduction

The prevalence of childhood overweight and obesity has increased dramatically during the last decades (1, 2). In Finland, 29% of boys and 18% of girls aged 7–12 years were overweight or obese at 2018 (3). Adiposity refers to increased body fat content which induces hazardous effects on health: adiposity increases cardiometabolic risk since childhood (4–7), tracks from childhood into adulthood (8, 9) and body fat excess in youth predicts metabolic syndrome, type 2 diabetes, atherosclerosis and premature mortality in adulthood (6, 10).

The prevalence of pediatric adiposity and its usefulness in predicting cardiometabolic diseases depend on the criteria i.e., the methods and the cut-points used (11–13). For example, a recent study showed that unhealthy body fat content was observed in 11–20% of girls and in 5–24% of boys when using waist-to-height ratio (WHtR), body mass index—standard deviation score (BMI-SDS) or body fat percentage (BF%) by dual-energy X-ray absorptiometry (DXA) as a diagnostic tool (11).

Pediatric adiposity has conventionally been assessed by BMI-SDS or BMI percentiles (14). A recent review concluded that anthropometric indicators, such as BMI percentiles or WHtR, have high capacity to discriminate children and adolescents with and without excess body fat (15), albeit BMI based measurements do not provide specific information on body fat content but only indirectly reflect true adiposity (16–18). Non-specificity means that normal weight individuals may have excess body fat and overweight individuals may be lean (19). From methodological point of view, this represents systematic misclassification which may explain the phenomenon called obesity paradox (20, 21). Therefore, more specific measures and criteria for adiposity are needed to identify individuals at increased risk of cardiometabolic diseases in clinical practice and at population level (5, 12, 18, 22).

WHtR represents an easily performed anthropometric measure to detect excess abdominal fat. Interestingly, albeit BMI may be a poor apriori index for adiposity, it has been proved to be a good predictor for premature mortality (23). It is therefore important to assess the ability of BMI-SDS and WHtR to identify children with increased cardiometabolic risk. Moreover, the capacity of less specific measures of adiposity needs to be compared with that of BF% measured by DXA, which represents a suitable reference method for children, or by bioelectrical impedance analysis (BIA), which is a mobile method and has sufficient trueness to be used in epidemiological purposes in children (11).

This study aimed to define diagnostic criteria for measures of adiposity, including BMI-SDS, WHtR and BF% assessed by BIA and DXA, to identify prepubertal girls and boys at increased cardiometabolic risk.

## Methods

### Study Design and Participants

The present analyses are based on the baseline data of the Physical Activity and Nutrition in Children (PANIC) study, which is a controlled physical activity and dietary intervention study in a population sample of primary school children from the city of Kuopio in Eastern Finland. The study was approved by the Research Ethics Committee of the Hospital District of Northern Savo in 2006 (statement number 69/2006) and was registered at ClinicalTrials.gov as NCT01803776. The children and their parents signed informed consent.

Altogether 736 children were invited, and 512 children 6–8 years of age participated in the baseline study in 2007–2009 (24). All except 11 children were Caucasian. The participants did not differ in age, sex distribution or BMI-SDS from all children who started the 1st grade in the city of Kuopio in 2007–2009 based on data from the standard school health examinations. Altogether 5 girls and 2 boys were excluded from the statistical analyses because of clinical puberty defined by Tanner stages (testicular size ≥4 ml in boys; breast development stage >B1 in girls) assessed by a physician (25, 26). Hence, only prepubertal children were included in the study because maturity process interacts with adiposity and cardiometabolic risk factors, such as insulin resistance (27–29). Moreover, 3 girls and 3 boys were excluded because no fasting blood samples were available, and 7 girls and 2 boys were excluded because of hemolysis in blood samples that hampered the measurement of fasting serum insulin. There were 217–221 girls and 240–249 boys with complete data for each measure of adiposity for the statistical analyses.

### Assessment of Body Size and Composition

Anthropometric measurements and segmental multi-frequency BIA (InBody 720®, Biospace Co, Seoul, Korea) were performed between 8 and 10 a.m. the children having fasted for 12 h and emptied the bladder. Body height was measured to nearest 0.1 cm using a wall-mounted stadiometer the children standing bare feet. Body weight was measured to nearest 0.1 kg using the BIA device the weight assessment integrated into the system. BMI was calculated as body weight (kg) divided by the square of body height (m). BMI-SDS and height-SDS were calculated according to national references (30). Waist circumference was measured after expiration at mid-distance between the bottom of the rib cage and the top of the iliac crest using inelastic tape to nearest 0.1 cm. WHtR was calculated by dividing waist circumference (cm) by body height (cm). DXA measurements were performed using the Lunar® device (Lunar Prodigy Advance, GE Medical Systems, Madison, WI, USA) and the Encore® software, Version 10.51.006 (GE Medical Systems, Madison, WI, USA). Total body fat mass and BF% were determined using BIA and DXA. The detailed assessment of body composition and agreement between BIA and DXA among prepubertal children have been reported elsewhere (11).

### Measurement of Cardiometabolic Risk Factors

Plasma high-density lipoprotein (HDL) cholesterol, triglycerides, glucose, alanine aminotransferase, gamma-glutamyl transferase, and high-sensitivity C-reactive protein (hs-CRP) and serum insulin were measured using 12 h overnight fasting samples as described in detail elsewhere (31). We excluded participants with hs-CRP values ≥5.0 mg/L to avoid confounding by subclinical infections (32), and hs-CRP values ≤0.3 mg/l were graded as 0.29 mg/l. Homeostatic Model Assessment for Insulin Resistance (HOMA-IR) was computed as (glucose^*^insulin)/22.5. Plasma uric acid was measured using an enzymatic colorimetric test (Roche Diagnostics CO, Mannheim, Germany). Plasma high-molecular-weight adiponectin was analyzed using an ELISA kit after specific proteolytic digestion of other multimeric adiponectin forms (Millipore, Billerica, MA, USA). Systolic and diastolic blood pressure were measured manually by a calibrated aneroid sphygmomanometer (Heine 130 Gamma G7®, HEINE Optotechnik GmbH & Co., Herrsching, Germany) 3 times at 2 min intervals in the sitting position after a 5 min rest. The average of 3 measurements was used in the analyses.

### Statistical Methods

The statistical analyses were performed using the SPSS software, version 21.0 (IBM SPSS Statistics, Armonk, NY, USA). The normality of the distributions of variables was defined using the Kolmogorov–Smirnov test. Medians (interquartile ranges, IQRs) were presented for variables with skewed distributions and means (standard deviations, SDs) for variables with normal distributions. Natural logarithmic transformation was used for WHtR, BF%, insulin, HOMA-IR, HDL cholesterol, triglycerides, alanine aminotransferase, gamma-glutamyl transferase and high-molecular-weight adiponectin and lg10 transformation for hs-CRP. Associations with *P*-values <0.05 were defined statistically significant. The independent samples *t*-test for parametric measures and the Mann-Whitney *U*-test for nonparametric measures were used to analyze differences in cardiometabolic risk factors between sexes.

We defined children having pediatric metabolic syndrome if they had at least 3 of its 4 components (1. hypertension: elevated systolic blood pressure or diastolic blood pressure, 2. impaired glucose control: increased fasting plasma glucose or HOMA-IR, 3. dyslipidemia: decreased plasma HDL cholesterol or increased plasma triglycerides, 4. abdominal obesity: increased waist circumference) using age- and sex-specific cut-points proposed by Ahrens and coworkers (33). We also composed a continuous cardiometabolic risk score (CMS) by searching the best combination of cardiometabolic risk factors to reflect metabolic disturbances associated with adiposity. For this purpose, we computed partial coefficients of correlation adjusted for age and sex between BF% assessed by DXA and a number of cardiometabolic risk factors among children below the median of BF% and above it (Table 1). BF% had statistically significant correlations with insulin, triglycerides, gamma-glutamyl transferase, uric acid, hs-CRP and HDL cholesterol among children in the upper half of BF% but not in the lower half which reflected metabolic disturbances associated with higher BF%. We therefore included these cardiometabolic risk factors in the CMS which represented the mean of Z-scores of cardiometabolic risk factors [(insulin + triglycerides + gamma-glutamyl transferase + uric acid + hs-CRP—HDL cholesterol)/6], and all approved components had equal weight on CMS. As waist circumference introduces analytical problems due to mathematical coupling in regard to other anthropometric measurements, it was not used as a potential component of the CMS.

**Table 1:** Partial coefficients of correlation between body fat percentage assessed by dual-energy X-ray absorptiometry and cardiometabolic risk factors among children below and above median of body fat percentage.

	**Body fat percentage**	
	**≤50th percentile**	**>50th percentile**
Blood pressure	0.09	0.02
Insulin	0.10	0.36^***^
Glucose	0.02	0.08
High-density lipoprotein cholesterol	0.01	−0.26^***^
Triglycerides	0.02	0.21^**^
High-sensitivity C-reactive protein	0.11	0.42^***^
Alanine aminotransferase	0.14^*^	0.20^**^
Gamma-glutamyl transferase	−0.03	0.34^***^
Uric acid	0.01	0.23^***^
High-molecular-weight adiponectin	0.07	−0.05

Blood pressure was assessed as a Z-score of average of diastolic and systolic blood pressure; Insulin was measured from fasting serum samples and other biomarkers were measured from fasting plasma samples; Partial coefficients of correlation were adjusted for age and sex;

* P <0.05;

** P <0.01;

*** *P <0.001*.

In primary analyses, we defined cut-points for the measures of adiposity associated with the risk of being in the 90th percentile of the CMS by the receiver operating characteristics (ROC) analysis. In secondary analyses, we defined cut-points for the measures of adiposity associated with the risk of having pediatric metabolic syndrome (33). The ROC analyses were performed separately for girls and boys, because girls had higher body fat content than boys (*P* < 0.001). To describe the diagnostic usefulness of the criteria for adiposity we defined sensitivity (true positive rate = true positive measurements / all positive cases, which includes true positive measurements and false negative measurements) indicating the ability to detect children with pediatric metabolic syndrome or increased CMS and specificity (true negative rate = true negative measurements / all negative cases, i.e., including true negative measurements and false positive measurements) indicating the ability to detect children without these outcomes. We defined the point where sensitivity is similar to specificity as the adiposity threshold indicating the overall ability of the measures of adiposity to detect children with and without pediatric metabolic syndrome or increased CMS. We also computed positive likelihood ratio [*LRpos: sensitivity/(1-specificity)*] that indicates the likelihood of correctly detecting children at increased cardiometabolic risk and negative likelihood ratio [*LRneg: (1-sensitivity)/specificity*] that indicates the likelihood of correctly excluding children at increased cardiometabolic risk. We aimed to find out cut-points representing LRpos >5 and LRneg <0.20 which refer to moderate usefulness of the measures of adiposity to identify children with and without increased cardiometabolic risk.

We graphed scatter plots with local regression curves using a Gaussian function and a 50% smoothing parameter to visualize non-linear associations of the measures of adiposity with the CMS and non-linear associations of BMI-SDS with WHtR and BF% assessed by BIA. Indeed, local regression curves were used to visualize points where cardiometabolic risk began to increase.

## Results

### Characteristics of Children

The characteristics of children are presented in Table 2. Mean (SD) CMS was −0.02 (0.48) in girls and −0.01 (0.54) in boys. Altogether 6 girls and 10 boys had pediatric metabolic syndrome, and 22 girls and 26 boys were above the 90th percentile of the CMS.

**Table 2:** Characteristics of children.

	**Girls**			**Boys**			***P*-value**
	**N**	**Mean/Median**	**SD/IQR**	**N**	**Mean/Median**	**SD/IQR**	
Age (years)	237	7.6	0.4	260	7.6	0.4	0.09
Height (cm)	237	127.7	5.6	260	129.7	5.6	<0.001
Height-SDS	237	0.11	0.98	260	0.17	1.04	0.51
Weight (kg)#	237	25.4	23.2–28.9	259	26.6	23.8–29.7	0.008
Waist circumference (cm)#	237	54.6	52.3–57.9	260	56.5	53.6–59.5	0.002
BMI-SDS	237	−0.20	1.05	259	−0.18	1.10	0.87
Waist-to-height ratio#	237	0.43	0.41–0.45	260	0.44	0.42–0.46	0.05
BF% by BIA#	237	16.5	12.8–21.1	259	13.2	10.1–18.3	<0.001
BF% by DXA#	232	20.5	17.3–26.9	251	15.1	11.4–21.8	<0.001
Insulin (uIU/ml) #	227	4.5	3.3–6.0	255	3.9	2.5–5.5	0.001
Glucose (mg/dL)	233	84.7	7.2	258	88.3	7.2	<0.001
HOMA-IR#	227	0.95	0.67–1.32	255	0.85	0.52–1.22	0.01
HDL cholesterol (mg/dL)#	234	60.2	52.9–66.4	258	61.8	55.2–69.1	0.04
Triglycerides (mg/dL)#	234	49.6	17.4–27.8	258	46.9	15.8–25.5	0.10
Hs-CRP (mg/l)#	226	0.29	0.29–0.62	252	0.29	0.29–0.48	0.01
ALT (U/l)#	234	18.0	15.0–20.0	258	18.0	15.0–21.0	0.79
GGT (U/l)#	234	11.0	10.0–13.0	258	12.0	10.0–13.0	0.57
Uric acid (mg/dL)	234	3.4	0.7	257	3.3	0.6	0.07
HMW-adiponectin (μg/ml)#	225	7.9	5.8–11.0	253	8.8	5.9–11.2	0.36
Systolic blood pressure (mmHg)	235	99.9	7.5	255	100.3	7.1	0.45
Diastolic blood pressure (mmHg)#	228	61.3	57.5–65.3	249	61.3	57.3–66.7	0.82
Mean blood pressure (mmHg)#	228	80.3	76.3–84.0	249	81.3	76.7–85.3	0.55

Insulin was measured from fasting serum samples and other biomarkers were measured from fasting plasma samples. The independent samples T-test for parametric measures and the Mann-Whitney test for non-parametric measures was used to analyze sex differences (P-value). Medians with interquartile ranges (IQR) were presented for non-parametric measurements and means with standard deviations (SD) were presented for variables with normal distributions. Mean blood pressure refers to arithmetic average of systolic and diastolic blood pressures. BF%, body fat percentage; ALT, alanine aminotransferase; BIA, bioimpedance analysis; BMI, body mass index; DXA, dual-energy X-ray-absorptiometry; GGT, gamma-glutamyl transferase; HDL, high density lipoprotein cholesterol; HMW, high molecular weight; HOMA-IR, Homeostatic Model Assessment for Insulin Resistance; hs-CRP, High sensitivity C-reactive protein; SDS, standard deviation score;

# *measurements with skewed distribution*.

### Diagnostic Ability of the Measures of Adiposity to Detect Increased Cardiometabolic Risk

**Figure 1:**
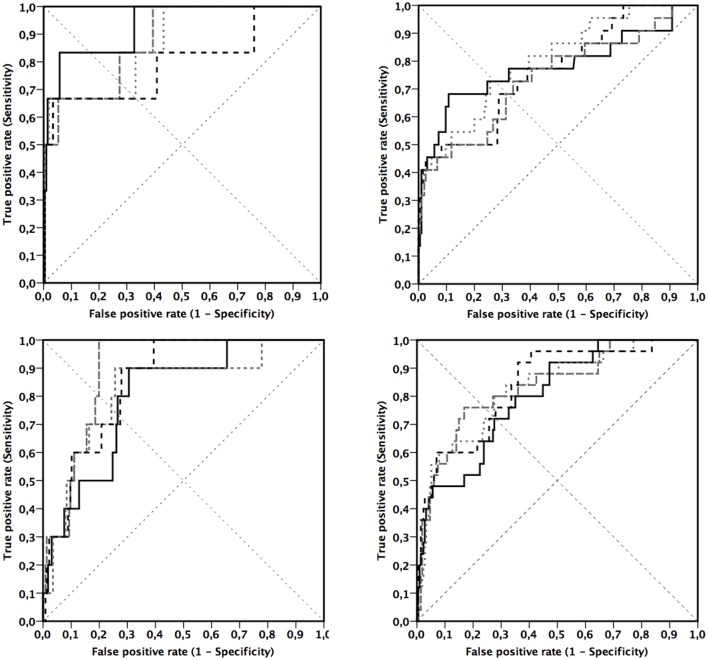
Receiver operating characteristic curves between the measures of adiposity and cardiometabolic risk in girls **(Upper)** and in boys **(Lower)**. Endpoint by cardiometabolic risk has been defined by pediatric metabolic syndrome (33) **(Left)** and using 90th **(Right)** percentile threshold for cardiometabolic score. Components of cardiometabolic score were serum insulin and plasma high-density lipoprotein cholesterol, triglycerides, gamma-glutamyl transferase, high-sensitivity C-reactive protein, and uric acid. Black lines represent waist-to-height ratio (___), body fat percentage by dual-energy X-ray absorptiometry (- - -), gray lines represent body mass index standard deviation score (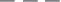), and body fat percentage by bioimpedance analysis (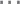).

All measures of adiposity had high areas under curve in girls and boys (Table 3). However, in girls WHtR (cut-point 0.445) and BF% assessed by BIA (cut-point 19.5%) had the highest point in the ROC-curve (Figure 1) with sensitivity being equal to specificity (0.73), which indicates that they were overall the best measures of adiposity to detect girls being above the 90th percentile of the CMS. Moreover, WHtR (sensitivity 0.68, cut-point 0.459) had the best ability to detect girls being above the 90th percentile of the CMS as indicated by the highest sensitivity related to LRpos > 5 (Table 3). Furthermore, WHtR was the best measure of adiposity to detect girls having pediatric metabolic syndrome as indicated by the measures shown in Table 3 and in Figure 1.

**Table 3:** Receiver operating characteristics and adiposity criteria for measures of adiposity among girls and boys by 90th percentile of the cardiometabolic risk score and by pediatric metabolic syndrome.

			**AUC (95% CI)**	**Cut–point (sensitivity; specificity)**		
				**Positive likelihood ratio >5**	**Point of the ROC–curve where sensitivity is equal to specificity**	**Negative likelihood ratio <0.20**
90th percentile of the cardiometabolic risk score	Girls	BMI–SDS	0.738^***^ (0.61–0.86)	1.01 (0.45; 0.91)	0.15 (0.68; 0.68)	*−1.57 (1.00; 0.09)*
		WHtR	0.778^***^ (0.65–0.91)	0.459 (0.68; 0.87)	0.445 (0.73; 0.73)	*0.396 (1.00; 0.09)*
		BF% by BIA	0.801^***^ (0.70–0.90)	25.1% (0.50; 0.90)	19.5% (0.73; 0.73)	15.2% (0.96; 0.38)
		BF% by DXA	0.763^***^ (0.65–0.87)	30.4% (0.50; 0.90)	23.3% (0.68; 0.68)	17.8% (0.96; 0.31)
	Boys	BMI–SDS	0.833^***^ (0.75–0.92)	0.76 (0.60; 0.88)	0.48 (0.76; 0.76)	−0.78 (0.96; 0.35)
		WHtR	0.799^***^ (0.71–0.89)	0.474 (0.48; 0.91)	0.447 (0.72; 0.72)	0.435 (0.92; 0.53)
		BF% by BIA	0.828^***^ (0.74–0.92)	19.5% (0.64; 0.87)	15.8% (0.72; 0.72)	13.8% (0.88; 0.60)
		BF% by DXA	0.839^***^ (0.76–0.92)	23.9% (0.60; 0.88)	19.1% (0.72; 0.72)	17.1% (0.88; 0.64)
Pediatric metabolic syndrome	Girls	BMI–SDS	0.880^**^ (0.75–1.00)	0.86 (0.67; 0.87)	0.37 (0.73; 0.73)	0.05 (1.00; 0.61)
		WHtR	0.930^***^ (0.83–1.00)	0.460 (0.83; 0.83)	0.460 (0.83; 0.83)	0.491 (0.83; 0.94)
		BF% by BIA	0.867^**^ (0.72–1.00)	24.9% (0.67; 0.87)	19.1% (0.67; 0.67)	17.3% (1.00; 0.57)
		BF% by DXA	0.798^*^ (0.57–1.00)	30.3% (0.67; 0.87)	23.7% (0.67; 0.67)	17.1% (1.00; 0.24)
	Boys	BMI–SDS	0.894^***^ (0.84–0.95)	0.60 (1.00; 0.80)	0.62 (0.80; 0.80)	0.604 (0.90; 0.80)
		WHtR	0.801^**^ (0.68–0.92)	0.487 (0.40; 0.92)	0.452 (0.74; 0.74)	0.447 (0.90; 0.69)
		BF% by BIA	0.821^**^ (0.68–0.96)	21.8% (0.60; 0.88)	18.0% (0.76; 0.76)	17.2% (0.90; 0.74)
		BF% by DXA	0.851^***^ (0.77–0.94)	25.6% (0.60; 0.88)	20.0% (0.73;0.73)	19.7% (0.90; 0.72)

*** P <0.001;

** P <0.01;

* *P <0.05; AUC, Area under Curve; BF%, body fat percentage; BIA, bioimpedance analysis; BMI–SDS, body mass index standard deviation score defined using national references (30); CI, Confidence Interval; DXA, dual–energy X–ray absorptiometry; ROC, Receiver operating characteristics; WHtR, waist–to–height ratio; There were 22 girls and 26 boys were above the 90th percentile of the cardiometabolic risk score and there were 6 girls and 10 boys who had pediatric metabolic syndrome (33)*.

In boys BMI-SDS (cut-point 0.48) had the highest point in the ROC-curve (Figure 1) with sensitivity being equal to specificity (0.76) (Table 3). However, BF% assessed by BIA (sensitivity 0.64, cut-point 19.5%) was a slightly more sensitive measure of adiposity to detect boys being above the 90th percentile of the CMS than BMI-SDS (sensitivity 0.60, cut-point 0.76) or BF% assessed by DXA (sensitivity 0.60, cut-point 23.9%) as indicated by the highest sensitivity related to LRpos >5. BMI-SDS was the best measure of adiposity to detect boys having pediatric metabolic syndrome as indicated by the measures shown in Table 3 and in Figure 1.

### Ability of Measures of Adiposity to Exclude Increased Cardiometabolic Risk

All measures of adiposity had a poor ability to exclude girls being above the 90th percentile of the CMS (Table 3). BF% assessed by BIA (cut-point 15.2%: specificity 0.38) was the best measure of adiposity to exclude girls being above the 90th percentile of the CMS. BF% assessed by DXA (cut-point 17.1%: specificity 0.64) was the best measure of adiposity to exclude boys being above the 90th percentile of the CMS as indicated by the highest specificity related to LRneg <0.20.

### Associations of the Measures of Adiposity With Cardiometabolic Risk

**Figure 2:**
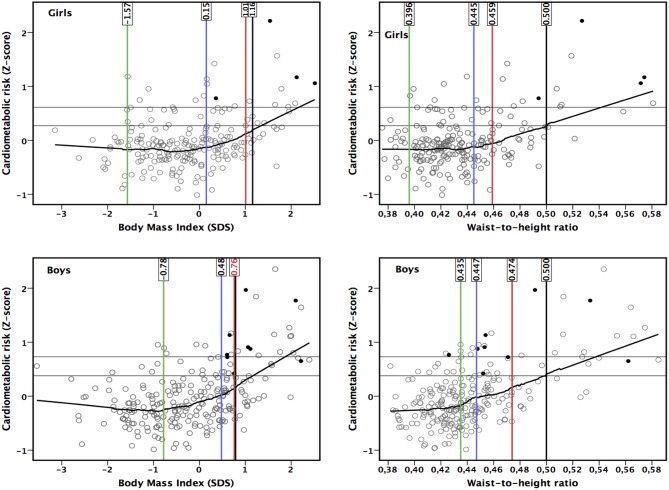
Associations of body mass index standard deviation score (SDS) and waist-to-height ratio with cardiometabolic risk score (components were serum insulin and plasma high-density lipoprotein cholesterol, triglycerides, gamma-glutamyl transferase, high-sensitivity C-reactive protein, and uric acid). Black dots represent children with metabolic syndrome (33). Horizontal lines describe the 80 and 90th percentiles for cardiometabolic risk score. Vertical lines represents the threshold by negative likelihood ratio (<0.20) (green line), the point where sensitivity equals with specificity (blue line), the threshold by positive likelihood ratio (>5) (red line), and the traditional national criteria (30) (black line).

**Figure 3:**
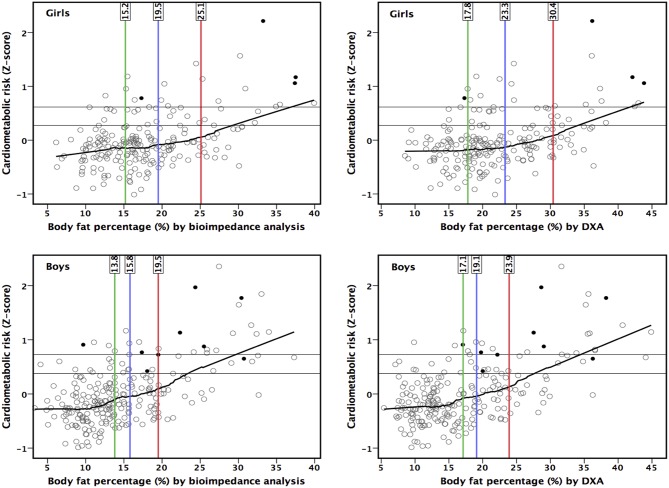
Associations of body fat percentage by bioimpedance analysis and by dual-energy X-ray absorptiometry (DXA) with cardiometabolic risk score (components were serum insulin and plasma high-density lipoprotein cholesterol, triglycerides, gamma-glutamyl transferase, high-sensitivity C-reactive protein and uric acid). Black dots represent children with metabolic syndrome (33). Horizontal lines describe the 80 and 90th percentiles for cardiometabolic risk score. Vertical lines represents the thresholds by negative likelihood ratio (<0.20) (green line), the point where sensitivity equals with specificity (blue line), and positive likelihood ratio (>5) (red line).

**Figure 4:**
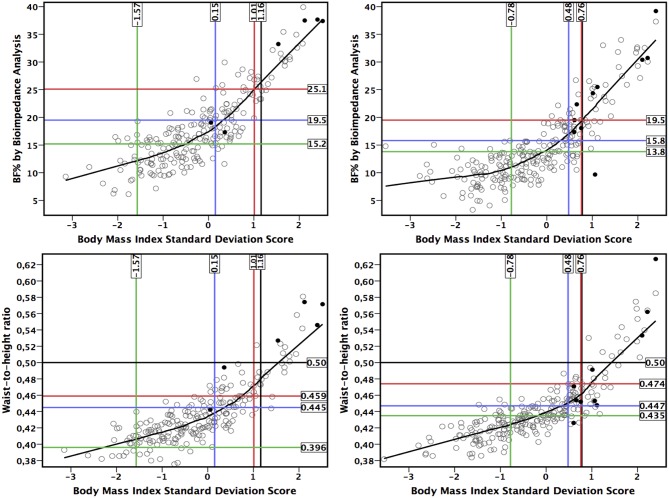
Association between body mass index standard deviation score, waist-to-height ratio and body fat percentage (BF%) by bioimpedance analysis in girls **(Left)** and in boys **(Right)**. Black dots represent children with metabolic syndrome (33). Vertical and horizontal lines represents: point where sensitivity equals with specificity (blue line), threshold by negative likelihood ratio (<0.20) (red line), threshold by positive likelihood ratio (>5) (green line), and the traditional national criteria (30) (black line).

The CMS started to increase from lower levels of measures of adiposity than suggested by LRpos >5 (Figures 2, 3). The point of ROC-curve where sensitivity is equal to specificity in girls and LRneg <0.20 in boys corresponded to a point in a local regression curve where the increase in the CMS started with increasing adiposity. Moreover, local regression curves showing non-linear associations of BMI-SDS with WHtR and BF% assessed by BIA suggest that the increases in WHtR and in BF% assessed by BIA vs. BMI-SDS steepen at higher levels of BMI-SDS (Figure 4).

## Discussion

Scientifically justified criteria for pediatric adiposity are needed to identify children who may have increased risk of metabolic syndrome, type 2 diabetes and cardiovascular disease in adulthood (5, 6, 34). We found that cardiometabolic risk among prepubertal children started to increase at a lower level of adiposity than the existing criteria for pediatric adiposity (14, 18, 30, 35–37). Our results suggest that WHtR of about 0.45 in girls and BMI-SDS of 0.48 in boys are the best overall cut-points for increased cardiometabolic risk. Moreover, measurements of adiposity have diagnostic capacity to be utilized as screening tool of elevated cardiometabolic risk. The measurement of BF% assessed by BIA or DXA offered no advantage over traditional anthropometric measures in detecting cardiometabolic risk in prepubertal children.

BMI-SDS of 1.01 in girls (specificity 91%) and 0.76 in boys (specificity 88%) had high specificity to identify prepubertal children being above the 90th percentile of the CMS. Therefore, the World Obesity Federation (i.e., former International Obesity Task Force) cut-point of BMI-SDS for girls (1.24) and boys (1.31) (14) and the Finnish overweight BMI-SDS cut-point for girls (1.16) (30) may not correspond to the definition of adiposity based on the thresholds of adiposity at which cardiometabolic risk begins to increase in our study. On the other hand, the Finnish overweight BMI-SDS cut-point for boys (0.78) (30) is quite similar to that observed in our study (0.76). The BMI-based cut-points of adiposity should have been lowered over the last decades (2, 17), because the average lean body mass has been decreasing in children (17). Our criteria of adiposity are based on the increase in CMS because the increase in fat content renders hazardous effects on cardiometabolic health and, therefore, our criteria are justified.

The results of a previous study (38) suggested that BMI would be a useful method to identify children with increased body fat content regardless of the methodological inability of BMI to differentiate between lean mass and fat mass. In line, we found that BMI-SDS had the best overall diagnostic ability in boys as indicated by the highest sensitivity being equal to specificity. Because BMI based measurements may reflect more strongly fat mass in children than in adults (2, 17, 22), more sophisticated and also more expensive measurements, such as body fat content assessed by DXA or BIA, may not offer advantage over BMI to identify children with adiposity-related metabolic abnormalities (16, 39). Indeed, BMI-SDS was associated more strongly with BF% and WHtR at higher BMI-SDS levels than in lower BMI-SDS levels. Moreover, we found that the acceleration in the increase of WHtR with increasing BMI-SDS in the local regression curves is consistent with the value for sensitivity being equal to specificity by the 90th percentile of the CMS in girls and in boys. This suggests that increased abdominal fat is particularly hazardous to cardiometabolic health. On the other hand, while the diagnostic ability for the measures of adiposity to exclude increased cardiometabolic risk was poorer than the ability to diagnose increased cardiometabolic risk, the local regression curves may demonstrate a rough physiological range for the measures of adiposity.

There is some evidence that WHtR may have better diagnostic and predictive ability as compared to BMI in children and adolescents (40, 41). The cut-points for WHtR of 0.445–0.460 in girls and 0.447–0.487 in boys in our study were lower than the previously proposed WHtR cut-point of 0.50 for abdominal obesity (16) but similar to the cut-points of 0.45–0.46 defined by Nambiar and coworkers (42). Moreover, the WHtR threshold detected by LRpos had appropriate sensitivity in girls but not in boys. Interestingly, the WHtR thresholds were rather similar in girls and boys, whereas the cut-points of BF% assessed by DXA (30%) and BIA (25%) were higher in girls than the cut-points of BF% assessed by DXA (25%) and BIA (20%) in boys. While the sex-related difference of 5% in BF% is fundamental and physiological (43), abdominal fat content, which reflects visceral fat tissue, seems to be equally dangerous for both sexes. This, in part, emphasizes the fact that increased abdominal fat, *per se*, is a strong predictor of cardiometabolic risk factors, such as insulin resistance (44–46) and endothelial dysfunction (44, 47, 48). Correspondingly, this may have particular importance in children because body fat tends to be more equally distributed in children as compared to adults (49). These findings suggest that girls may have better ability to tolerate a higher BF% but not abdominal adiposity for their cardiometabolic health than boys. These cut-points of BF% assessed by DXA and BIA to detect girls and boys at increased cardiometabolic risk are similar to those published previously (35–37). Moreover, the observed 5% difference between the thresholds of BF% assessed by DXA and by BIA represents a bias between these methods (11).

We used a specifically optimized CMS as an indicator for increased cardiometabolic risk instead of single risk factors, such as fasting serum insulin, because adiposity induces the clustering of cardiometabolic risk factors (1, 5, 6, 33, 34) and because the use of a continuous risk score may increase statistical power and may be less prone to errors than dichotomous variables for increased risk (34, 50–53). Although continuous scores for metabolic syndrome may not offer predictive advantage over dichotomous definitions (54), no optimal dichotomous threshold has been defined either (33, 55, 56). Furthermore, we performed analyses using the presence of pediatric metabolic syndrome (33) representing a clinical endpoint in the ROC analysis, and these results were quite similar as compared to the results by the 90th percentile of the CMS.

We composed a continuous CMS by searching the best combination of cardiometabolic risk factors to reflect metabolic disturbances associated with adiposity. For this purpose, we correlated BF% assessed by DXA and a number of cardiometabolic risk factors among children below the median of BF% and above it. The observed cardiometabolic risk factors for CMS are also biologically justified. The likelihood of increased triglycerides and decreased HDL cholesterol and decreased insulin sensitivity as a consequence of obesity has been shown in children (57, 58). Furthermore, insulin resistance is associated with increased plasma free fatty acids and hypertension (59, 60). Insulin resistance and increased circulating free fatty acids increase triglyceride synthesis and secretion in the liver which introduces an atherogenic lipoprotein profile (61). In line, increased plasma liver enzymes, such as gamma-glutamyl transferase, have been found to be indicators of liver fat accumulation and have been associated with increased cardiometabolic risk in children (31). Increased plasma hs-CRP, a measure of chronic low-grade inflammation, has been associated with obesity and other cardiometabolic risk factors, such as insulin resistance, increased plasma triglycerides and uric acid and decreased plasma HDL cholesterol in children (58, 62). Moreover, abnormal uric acid metabolism related to insulin resistance may be due to a hyperinsulinemia-mediated decrease in uric acid clearance by kidneys or due to increased serum uric acid reflecting disturbed purine metabolism (61, 63). Hyperuricemia has also been an independent predictor for hypertension in children and adolescents (64). Our CMS did not include components of blood pressure and fasting plasma glucose unlike the criteria of pediatric metabolic syndrome (33) or traditionally used metabolic risk scores (31). Hypertension is a major risk factor for global burden of diseases (65) and can be described as a silent killer (66). However, elevated blood pressure pathophysiologically reflects consequence of arterial stiffness. Moreover, increased fasting plasma glucose is secondary state due to insulin resistance. This may explain why these measures associated with adiposity were not included in CMS in our study.

A strength of our study is that multiple measures of adiposity and different cardiometabolic risk endpoints were available, which enabled us to assess the diagnostic usefulness of different adiposity criteria. Moreover, our population-based cohort included more girls and boys in the target age group than previously studied populations (35–37). However, the observed criteria were produced for Finnish prepubertal children, and these criteria cannot be applied in other age groups or other ethnic backgrounds without further studies. Therefore, the associations of the measures of adiposity with cardiometabolic risk should also be assessed in other age groups. Unfortunately, we had only 6 girls with pediatric metabolic syndrome, which reduced the statistical power of ROC-analysis to discriminate pediatric metabolic syndrome in girls. However, sample size calculation suggested that the number of girls with pediatric metabolic syndrome was sufficient in regard to ROC-analysis.

The findings of our study offers practical applications and clinically as well as scientifically interesting implications. This study provides method- and sex-specific adiposity criteria based on cardiometabolic risk and provides diagnostic usefulness for different measures of adiposity. This helps to choose the most useful methods for clinical practice and promotes epidemiological comparisons. Observed AUCs suggest that the measurement of adiposity can be used to screen elevated cardiometabolic risk in prepupertal children (67). The screening of elevated cardiometabolic risk can be done without body composition specific methods. This means that WHtR and BMI-SDS can well be applied as diagnostic tools for the screening purposes. On other hand, pediatric metabolic syndrome could be diagnosed by excellent accuracy by assessing body fat excess (67). However, in the clinical practice, the additional value of body compartment specific methods to define adiposity may be minimal. Moreover, cardiometabolic risk seems to begin to arise at a lower level of adiposity than suggested by traditional ROC-analyses. Therefore, local regression curves or similar kinds of analyses should be prioritized. Local regression curves, instead of crude statistical allocation, may be more useful to assess biological variation of adiposity introduced risks and individual tolerance to excess body fat.

## Conclusions

The observed AUCs of all measures of adiposity indicate that their diagnostic capacity to discriminate children with pediatric metabolic syndrome was excellent. Correspondingly, the observed diagnostic capacity to identify children with CMS means that measures of adiposity can be used to screen children with elevated cardiometabolic risk. A WHtR of about 0.45 in girls and a BMI-SDS of 0.48 in boys may be the most valid and useful overall criteria to diagnose elevated cardiometabolic risk. BF% assessed by BIA or DXA offered no advantage over anthropometric measures to detect prepubertal children with increased cardiometabolic risk. Cardiometabolic risk began to increase well below traditional criteria for adiposity. The adiposity thresholds detected allow the early identification of prepubertal children with increased cardiometabolic risk and targeting additional measurements and lifestyle interventions to these children. To increase understanding of the pathophysiology of increased cardiometabolic risk due to adiposity in children and adolescents, future studies should evaluate the concordance and divergence of individual cardiometabolic risk factors induced by body fat excess.

## Data Availability

The datasets generated for this study are available on request to the corresponding author.

## Ethics Statement

This study was carried out in accordance with the recommendations of the Research Ethics Committee of the Hospital District of Northern Savo in 2006 (declaration number 69/2006) and was registered at ClinicalTrials.gov as NCT01803776. The children and their parents signed informed consent, and all subjects gave written informed consent in accordance with the Declaration of Helsinki. The protocol was approved by the Research Ethics Committee of the Hospital District of Northern Savo in 2006.

## Author Contributions

TT collected data, carried out the analyses, conceptualized and designed the study, drafted the initial manuscript, and reviewed and revised the manuscript. TL, JJ, and TAL conceptualized and designed the study and reviewed and revised the manuscript. VL, AV, and A-ME collected data and critically reviewed the manuscript. DL critically reviewed the manuscript and edited language of the manuscript. All authors approved the final manuscript as submitted and agree to be accountable for all aspects of the work.

### Conflict of Interest Statement

TT is employed by Sense4Health Ltd. The remaining authors declare that the research was conducted in the absence of any commercial or financial relationships that could be construed as a potential conflict of interest
